# Understanding the Impact of Active-to-Passive Area Ratio on Deformation in One-Dimensional Dielectric Elastomer Actuators with Uniaxial Strain State

**DOI:** 10.3390/ma16216897

**Published:** 2023-10-27

**Authors:** Hans Liebscher, Markus Koenigsdorff, Anett Endesfelder, Johannes Mersch, Martina Zimmermann, Gerald Gerlach

**Affiliations:** 1 Institute of Solid-State Electronics, Faculty of Electrical and Computer Engineering, TUD Dresden University of Technology, Mommsenstraße 15, 01069 Dresden, Germany; hans.liebscher@tu-dresden.de (H.L.); markus.koenigsdorff@tu-dresden.de (M.K.); gerald.gerlach@tu-dresden.de (G.G.); 2 Fraunhofer Institute for Material and Beam Technology IWS, Winterbergstraße 28, 01277 Dresden, Germany; anett.endesfelder@iws.fraunhofer.de (A.E.); martina.zimmermann@iws.fraunhofer.de (M.Z.); 3 Institute of Materials Science, Faculty of Mechanical Science and Engineering, TUD Dresden University of Technology, Helmholtzstraße 7, 01069 Dresden, Germany; 4 Institute of Measurement Technology, Department of Mechatronics, Johannes Kepler University Linz, Altenberger Straße 69, 4040 Linz, Austria

**Keywords:** dielectric elastomer actuator, fiber–elastomer composite, active-to-passive area ratio, unidirectional actuator, electro-mechanical transducer, lumped-parameter model, equivalent circuit, anisotropy

## Abstract

There is increasing interest in the use of novel elastomers with inherent or modified advanced dielectric and mechanical properties, as components of dielectric elastomer actuators (DEA). This requires corresponding techniques to assess their electro-mechanical performance. A common way to test dielectric materials is the fabrication of actuators with pre-stretch fixed by a stiff frame. This results in the problem that the electrode size has an influence on the achievable actuator displacement and strain, which is detrimental to the comparability of experiments. This paper presents an in-depth study of the active-to-passive ratio with the aim of investigating the influence of the coverage ratio on uniaxial actuator displacement and strain. To model the effect, a simple lumped-parameter model is proposed. The model shows that the coverage ratio for maximal displacement is 50%. To validate the model results, experiments are carried out. For this, a rectangular, fiber-reinforced DEA is used to assess the relation of the coverage ratio and deformation. Due to the stiffness of the fibers, highly anisotropic mechanical properties are achieved, leading to the uniaxial strain behavior of the actuator, which allows the validation of the one-dimensional model. To consider the influence of the simplifications in the lumped-parameter model, the results are compared to a hyperelastic model. In summary, it is shown that the ratio of the active-to-passive area has a significant influence on the actuator deformation. Both the model and experiments confirm that an active-to-passive ratio of 50% is particularly advantageous in most cases.

## 1. Introduction

In its conventional configuration, a dielectric elastomer actuator (DEA) comprises a deformable capacitor structure with two compliant electrodes separated by a dielectric elastomer (DE). When a voltage is applied across the electrodes, the DE is compressed in the electric field direction, causing it to expand biaxially in-plane [1,2]. This expansion can be significantly increased by applying pre-stretch to the DE. To preserve the pre-stretch, different approaches exist, such as biasing mechanisms for instance a fixed mass, pre-tensioned linear springs, or negative bias springs [3,4]. Another common approach is bonding the pre-stretched DE to a rigid carrier frame. The fixed DEA can then be integrated into mechanical systems. Additionally, this serves as an experimental setup to compare the electro-mechanical properties of different DE films [5].

Fixing the pre-stretch with a frame, as opposed to using biasing mechanisms, poses the problem that the ratio between the active (electrode coating) and passive (no electrode coating) area in the actuator influences the achievable electro-active strain. For example, circular DEAs, or so-called dot actuators, are used to assess the electro-mechanical performance of DEs by evaluating the electrode strain during actuation. These actuators are composed of a pre-stretched DE membrane fixed on a circular frame and centered electrodes with recommended radii of one tenth to one third of the total DE radius. Such a ratio is supposed to have the effect that the pre-stretch behaves similarly to a constant biasing force [6]. However, this ratio specification is not sufficient as a precise standard, since the wide range of electrode sizes means that actuation strain studies by different research groups are difficult to compare. For instance, there are various investigations on the commercial DE VHB 4910, in which the ratio of the electrode radius to the inner radius of the carrier frame (total DE radius) differs significantly [7,8,9,10]. In various articles that have been published in the last two years, the coverage ratio is not considered and is chosen arbitrarily [11,12,13].

The influence of the active-to-passive ratio has already been analyzed in DEA-driven mechanical systems. Rosset et al. investigated the uniaxial displacement of a rigid platform attached to a DE membrane that was pre-stretched in two perpendicular directions in-plane and clamped onto a frame. The platform was placed at the border between the electrode area and passive region. Due to its rigid properties, the platform led to a local uniaxial strain state in-plane during DEA operation. To achieve maximal uniaxial in-plane displacement, an optimal ratio between the active and passive areas of 50% was found for this DEA configuration [14]. In another study, carried out on a similar DEA assembly, Rosset et al. established a non-linear analytical model based on the hyperelastic Gent model to calculate the mechanical energy stored in the active and passive zones [15]. The model predicted an optimum active-to-passive ratio of 46%. However, this model does not allow simple analytical relationships between deflection and geometrical and material parameters.

## 2. Lumped-Parameter Model

**Figure 1:**
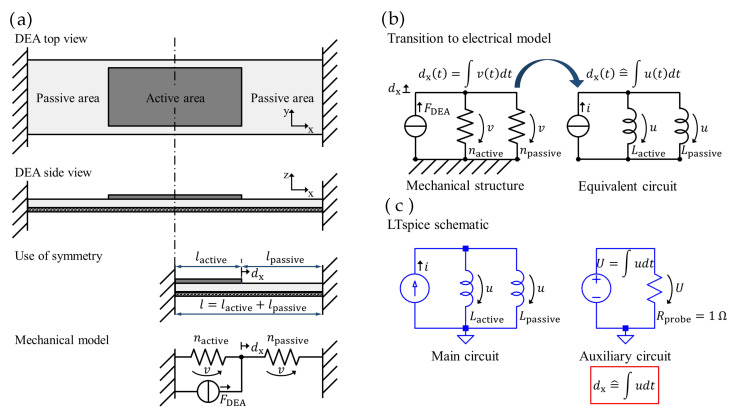
Derivation of the equivalent circuit model: (**a**) lumped-parameter model for unidirectional DEA behavior, (**b**) equivalent circuit representing an ideally elastic DEA, (**c**) LTspice^®^ schematic.

To predict the electrically induced electrode strain $s_{x}$ and displacement $d_{x}$ in the positive *x*-direction, a lumped-parameter model of a rectangular strip DEA is developed and implemented as an equivalent circuit model in a simulation program with integrated circuit emphasis (SPICE). This model is based on the assumption of an ideal DEA. It assumes that the relative permittivity of the DE film remains constant with strain and that the material is incompressible ($\lambda_{x}\lambda_{y}\lambda_{z}=1$). The DE material is considered to be linear-elastic around the operating point defined by the pre-stretch. Furthermore, infinite stiffness is assumed in the y-direction to enable unidirectional deformation in the x-direction. The actuator is pre-stretched and clamped on both sides, with the active area positioned symmetrically between the surrounding passive regions. Taking advantage of the DEA’s symmetry, the geometry is halved (Figure 1a). The active-to-passive area ratio *B* is defined by

(1) $$B=\frac{l_{active}}{l},$$

where *l* is half of the clamped actuator length and $l_{active}$ is half of the top electrode length. Both areas are described by the spring compliances $n_{active}$ and $n_{passive}$ with the velocity *v*, whereby the active area corresponds to a tension spring and the passive area to a compression spring. The respective compliances are given by

(2) $$n_{active}=\frac{l_{active}}{YA_{cross}},$$

and

(3) $$n_{passive}=\frac{l_{passive}}{YA_{cross}},$$

where *Y* is the Young’s modulus at the respective operating point and $A_{cross}$ is the stretch-state-dependent cross-sectional area of the strip. The DEA force is represented by the load source having a value defined by

(4) $$F_{DEA}=\sigma_{Maxwell}A_{DEA}=\varepsilon_{0}\varepsilon_{r}\frac{U_{HV}^{2}}{z_{DE}}w\lambda_{pre},$$

with the Maxwell stress $\sigma_{Maxwell}$, which is induced by the applied high voltage $U_{HV}$ and the specific part of the cross-sectional area $A_{DEA}$ where the Maxwell stress is induced. Furthermore, $z_{DE}$ is the initial DE layer thickness, *w* the width of the top electrode, $\lambda_{pre}$ the pre-stretch, $\varepsilon_{0}$ the permittivity in free space, and $\varepsilon_{r}$ the relative dielectric constant of the DE. Consequently, the following analytical solution can be obtained for the displacement $d_{x}$:

(5) $$d_{x}=B\left(1-B\right)\frac{l}{YA_{cross}}F_{DEA}.$$

The corresponding strain $s_{x}$ can then be calculated by

(6) $$s_{x}=\frac{d_{x}}{l_{active}}.$$

The analytical solution for the displacement $d_{x}$ is obtained from Equation (5) and the maximum displacement occurs when $B=\frac{1}{2}$. This indicates that the optimum coverage rate with respect to $d_{x}$ is half of the surface area. Translated to the mechanical model of Figure 1a, this means that the largest displacement $d_{x}$ due to the DEA’s force will occur exactly when the two compliances $n_{active}$ and $n_{passive}$ are equal.

To transform the mechanical structure into an equivalent electrical circuit, electro-mechanical analogies are utilized based on Firestone’s analogy [16] as listed in Table 1. Figure 1b illustrates the transition from the mechanical system to the electrical network, which has an isomorphic structure.

To simulate the unidirectional actuator behavior, the network analysis and synthesis program LTspice^®^ (Version 17.0.33.0, Analog Devices Inc., Wilmington, NC, USA) is used. Figure 1c shows the schematic, consisting of a main circuit (the equivalent circuit) and an auxiliary circuit (used to integrate the nodal voltage *u*). The integral of the nodal voltage *u* corresponds to the displacement $d_{x}$, similar to the way in which the integral of the velocity *v* corresponds to the displacement $d_{x}$ in the mechanical domain. This approach allows for the efficient simulation and analysis of the actuator’s electro-mechanical behavior.

## 3. Materials and Methods

**Figure 2:**
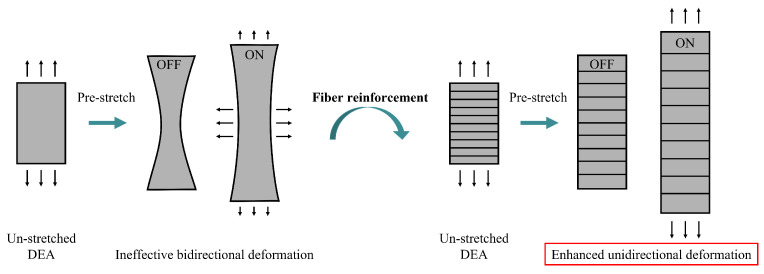
Use of fiber reinforcement for enhanced unidirectional DEA deformation.

To obtain uniaxial actuation behavior, various approaches have been reported in the literature. These include using 3D-printed patterns coupled to the DEA structure [17,18], employing wrinkled DE films and electrodes [19,20], or rolling DEAs into cylindrical-shaped actuators [21]. Additionally, DE materials have been modified with particle fillers to create composites with locally adjusted stiffness for bending and linear displacement applications [22,23]. Another technique involves using locally hardened DE films that have been cured only in selected areas to break the symmetry of deformation in-plane [24]. Incorporating stiff fibers into DE layers [25] or actuator electrodes [26] offers a promising approach to achieve DEA structures with a high degree of anisotropy and can lead to enhanced unidirectional deformation (Figure 2).

To manufacture these DEAs, CNT-based sheets [27] or carbon-fiber-based textiles [26,28] are functionalized as electrodes, allowing for the assembly of strip actuators [29,30]. To investigate the influence of the active-to-passive ratio in the one-dimensional case, in this work, fiber-reinforced strip actuators are used.

### 3.1. Manufacturing

**Figure 3:**
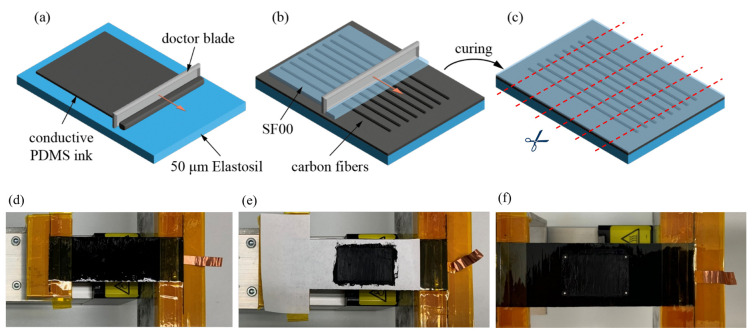
Manufacturing process of the uniaxial DEA. (**a**) Doctor blading of conductive PDMS ink onto Elastosil^®^ film. (**b**) Bonding of the unidirectional carbon fibers through infiltration on top of the stack. (**c**) Cutting of the composite into strips. (**d**) Clamping the strip into uniaxial pre-stretch device. (**e**) Application of carbon grease electrode through mask on pre-stretched strip. (**f**) Placing optical tracking markers in corners of carbon grease electrode.

The unidirectional aligned carbon fibers (ZOLTEX PX35 50k, R&G Faserverbundwerkstoffe GmbH, Waldenbuch, Germany) provide mechanical anisotropy, suppressing the deformation in the fiber direction and therefore reducing the active-to-passive ratio problem to one dimension. Furthermore, they are integrated into one of the electrodes, improving its electrical conductance. The DEA manufacturing process consists of six steps and is illustrated in Figure 3.

*Step 1. Creating the first electrode (bottom electrode).* To improve the homogeneity of the conductivity in the fibrous electrode, a base layer of electrically conductive silicone is applied to a 50 µm thick DE of ELASTOSIL^®^ Film 2030 250/50 (Wacker Chemie AG, Munich, Germany) with a doctor blade. Additionally, this layer serves as a protective layer for the dielectric film to prevent damage caused by any potentially broken fibers. The conductive ink is produced by mixing polydimethylsiloxane (PDMS, SF00, Silikonfabrik, Ahrensburg, Germany) with 7% carbon black particles (VULCAN^®^ XC72R, Cabot Corporation, Billerica, MA, USA) and DOWSIL^TM^ OS-10 (Dow Silicones Belgium, Seneffe, Belgium) in a speed mixer (Thinky ARE-250, Thinky Corporation, Tokyo, Japan). After the film application, the sample is transferred to a 70 °C oven to cure and evaporate all remaining OS-10.

*Step 2. Bonding the fibers.* When the curing process is finished, the carbon fiber fabric is bonded to the conductive PDMS electrode. This is achieved by infiltrating the dry carbon fibers on top of the cured conductive PDMS layer by running a doctor blade along the fibers to remove any excess material. The stack is then left to cure at room temperature for 2 h and then transferred to a 70 °C oven to finish the curing process. This reduces the number of air bubbles and dry spots in the composite, hence improving the fiber–matrix bond across the whole structure. This is crucial, as dry spots of uninfiltrated fibers close to the composite edge might act as starting points for delamination in the stack.

*Step 3. Cutting the actuators.* For the study, three strip actuators are manually cut to a size of 85 mm × 30 mm with a scalpel. Before the second electrode is applied, the fixing yarns that held the carbon fibers in place for better handling and infiltration are manually removed with pliers.

*Step 4. Clamping the actuator.* The fiber-reinforced elastomer strips are fixed one at a time with polyimide tape, tesa 51408 (tesa SE, Norderstedt, Germany), on a self-built uniaxial stretching device. The initial un-stretched clamped length of each strip is set to 57 mm. Uniaxial pre-stretch ratios of $\lambda_{pre}$ of 1.2, 1.3, and 1.4 are applied, respectively.

*Step 5. Applying the second electrode (top electrode).* To enable the investigation of different active-to-passive ratios, the size of the second electrode has to be varied. Therefore, carbon grease made from part-A silicone ExSil^®^50 (Gelest Inc., Morrisville, NC, USA) and 11 phr of carbon black PRINTEX^®^ XE-2B (Orion Engineered Carbons GmbH, Eschborn, Germany) is applied with masks and a spatula on the respective pre-stretched strip. The top electrode width is set always to 20 mm and surrounded by a safety gap of 5 mm to prevent short circuits with the bottom electrode in the edge region. For each strip actuator, the active-to-passive ratio is varied in the range from 10% to 90% in 5% increments by changing the surface area of the top electrode, by increasing the length of the top electrode while keeping the width of 20 mm constant. The respective pre-stretch is also kept constant during the electrode deposition over the whole ratio range.

*Step 6. Placing of the optical tracking markers.* White-colored circular tracking markers with a diameter of 1 mm (MTT Measurement Technology Trade GmbH, Dresden, Germany) are placed in the corners of the top electrode.

### 3.2. Electro-Mechanical and Mechanical Characterization

**Figure 4:**
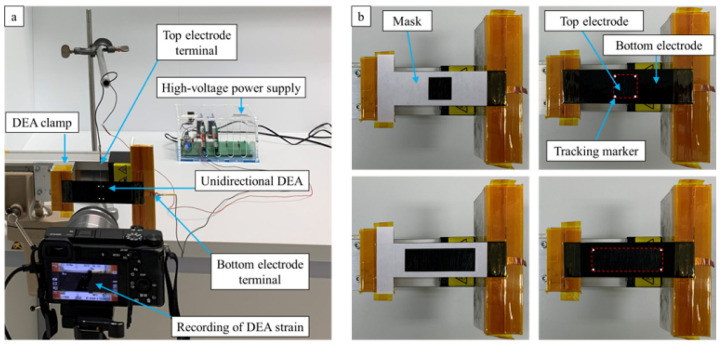
(**a**) Measurement setup for DEA strain. (**b**) Manufacturing of different active-to-passive ratios by variation of top electrode size, ratio of 25% (upper) and ratio of 75% (lower).

As described in Section 3.1, the influence of the active-to-passive ratio is studied on three differently pre-stretched DEAs. Figure 4a depicts the experimental setup used to drive a strip DEA and observe its electrically induced deformation. The top electrode’s adjustment during the electro-mechanical characterization is shown in Figure 4b for examples with active-to-passive ratios of 25% and 75%. To investigate the electrical actuation strain and passive mechanical recovery, a video of the top electrode area is recorded over a period of 25 s at each active-to-passive ratio. The video capturing is carried out using a compact camera (Sony α6400) with a macro lens (Sony SEL30M35) at a speed of 100 frames per second. The distance between the lens and the top electrode region amounts to 12.5 cm. During the first 5 s of the video recording, the actuator is in a zero-voltage state to detect the initial positions of the tracking markers, which corresponds to the initial top electrode size. Then, a constant voltage level of 2.5 kV is applied for 10 s using a high-voltage power supply (Peta-pico-Voltron) [31]. This leads to the uniaxial stretching of the actuator and thus the primarily horizontal displacement of the tracking markers. Subsequently, the voltage is switched off and the video recording continues for 10 s to track the recovery behavior of the strip DEA.

The video processing and the pixel-based extraction of the top electrode strain are performed in MATLAB. The MATLAB function “imfindcircles” is used to detect the four white tracking markers as circular objects in the images and to return their center coordinates as well as radii. In order to evaluate the horizontal strain $s_{x}\left(t\right)$ of the top electrode over the measuring time, in each video frame *n*, the center x-positions of the two tracking markers on the right and left are averaged, respectively. Then, the distance $\Delta x\left[n\right]$ between the two averaged positions can be determined and the strain $s_{x}\left[n\right]$ can be calculated with respect to the averaged distance that was obtained for the first frame $\Delta x\left[1\right]$ using the equation

(7) $$s_{x}\left[n\right]=\frac{\Delta x\left[n\right]}{\Delta x\left[1\right]}-1.$$

In a similar way, the vertical strain $s_{y}\left[n\right]$ is calculated for each frame *n* using the center y-positions of the tracking markers. The consideration of both strains $s_{x}\left(t\right)$ and $s_{y}\left(t\right)$ enables the evaluation of the anisotropic properties of the fiber-reinforced strip DEAs. The values of the time-dependent strain $s_{x}\left(t\right)$ are averaged in the range of 1 s to 4 s and 6 s to 14 s, to obtain the strain values for the “voltage off” and “voltage on” state, respectively, which are used to calculate the strain $s_{x}$ for each active-to-passive ratio.

After the optical strain measurements, uniaxial tensile tests of the DEA composites are carried out in a universal testing machine (Inspekt table 50 k, Hegewald und Peschke MPT GmbH, Nossen, Germany) with a 10 N load cell. The composite samples are fixed with pneumatic clamps. The test parameters are based on the DIN 53504 standard [32] with a strain rate of 1 mm/s. All samples have a rectangular shape with a clamping length of 60 mm and a width of 30 mm. The thickness of the un-stretched sample is measured using an incremental probe (IKF 10, Feinmess Suhl GmbH, Suhl, Germany) with a flat circular measuring insert (diameter of 4 mm) and a display unit (PU 11, Feinmess Suhl GmbH, Germany). Per sample, 20 readings are taken. The averaged thickness $t_{DEA,initial}$ is used for the calculation of the engineering stress.

## 4. Results

### 4.1. Electro-Mechanical Characterization

**Figure 5:**
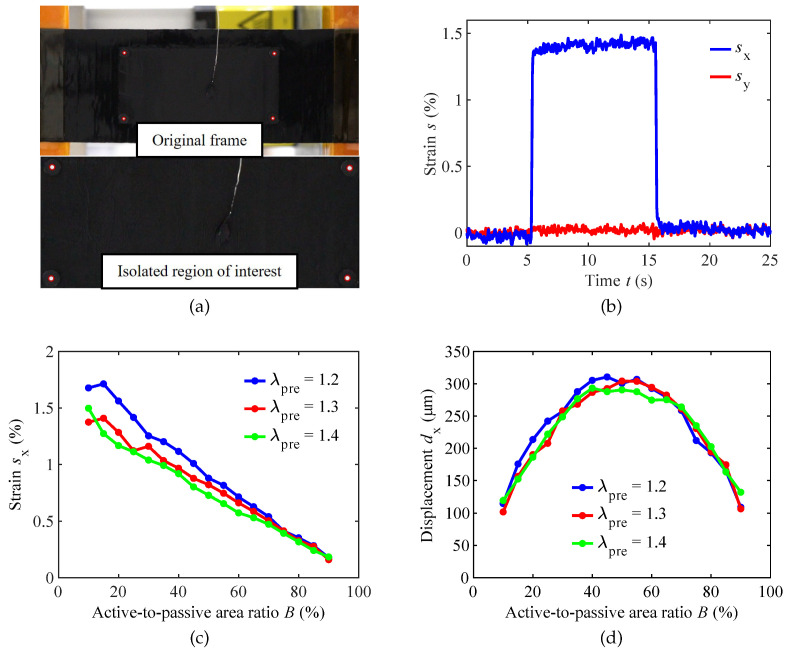
Measurement results. (**a**) Detection of the tracking points in an exemplary video frame and (**b**) time-dependent strain of an actuator with $\lambda_{pre}$ = 1.2 and *B* = 25% at 2.5 kV in *x*-direction and *y*-direction. Dependence of (**c**) measured electro-active strain $s_{x}$ and (**d**) corresponding displacement $d_{x}$ on the active-to-passive ratio *B*.

The captured video of the actuator is evaluated with the MATLAB script described in Section 3.2. As depicted in Figure 5a, the algorithm detects the tracking points in each video frame. Figure 5b illustrates the results of the optical strain measurements in the *x*- and *y*-direction conducted on one of the three pre-stretched DEAs under investigation. As expected, due to the reinforcing carbon fibers, the active strain $s_{y}$ is negligible. In contrast, $s_{x}$ increases once the voltage is applied after 5 s and returns to its initial state after 10 s. From the series of measurements for varying coverage ratios and pre-stretches, the results depicted in Figure 5c are derived.

The experimental findings reveal a consistent linear decrease in electrically induced strain as the active-to-passive area ratio increases for the tested DEAs. Remarkably, at lower active-to-passive ratios, noticeable variations in actuation strain are observed among the different pre-stretch levels of the DEAs. This suggests that the pre-stretch level significantly affects the actuation performance when the active area is relatively smaller than the passive one. However, as the active-to-passive ratio approaches 90%, these differences in actuation strain between the various pre-stretch levels become less pronounced. An unexpected finding is that the DEA with the lowest pre-stretch level exhibits the most substantial electro-active strain. This result indicates that higher pre-stretch levels may cause the stiffening of the dielectric material, leading to reduced actuation performance.

To calculate the displacement $d_{x}$, the strain $s_{x}$ is multiplied by the pre-stretch-dependent electrode length $l_{active}$. The results are presented in Figure 5d. It is observed that all tested samples follow a similar parabolic curve across the entire measured range of active-to-passive area ratios. Consistent with expectations and with the results from the simplified model of Equation (5), the maximum displacement occurs at approximately a 50% active-to-passive area ratio for all samples. This finding confirms that maintaining a balanced 1:1 ratio between the active and passive lengths is crucial in optimizing the actuation performance of the DEAs, regardless of the varying levels of pre-stretch applied.

### 4.2. Comparison of the Lumped-Parameter Model Results

**Figure 6:**
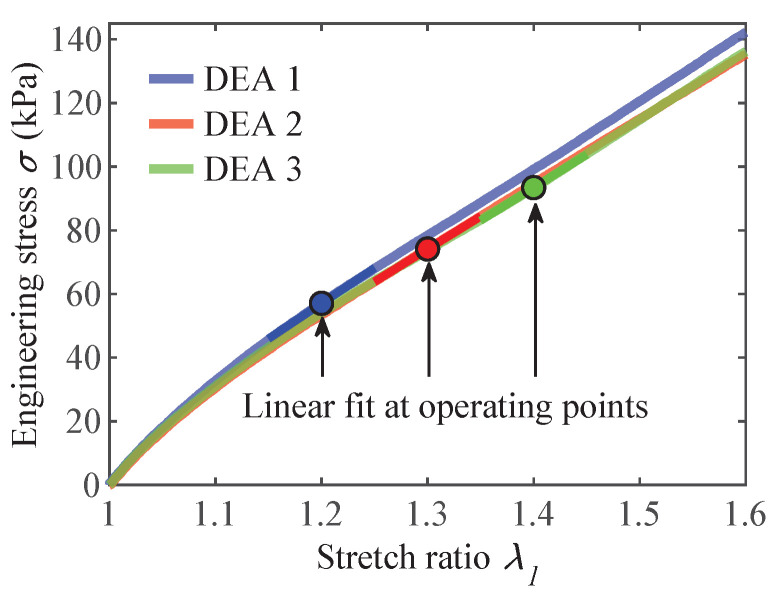
Stress–strain measurement of the fiber-reinforced DEA perpendicular to fiber direction with linear fits around operating points to obtain Young’s modulus *Y*.

To simulate the actuators with the lumped-parameter model, it was necessary to determine the Young’s modulus of the tested samples. As explained in Section 3.2, the same samples used for the optical actuation strain investigations underwent uniaxial tensile tests. Figure 6 illustrates the results of these tests, along with the linear fit of the stress–strain curve at the corresponding operating point $\lambda_{pre}$. The Young’s modulus values and initial sample thickness of each actuator are provided in Table 2.

Surprisingly, contrary to the results shown in Figure 5c, increasing the pre-stretch did not lead to the stiffening of the material. Instead, it resulted in a slight reduction in mechanical stiffness. Consequently, one would expect higher actuation strains with higher pre-stretch, but this was not observed. The reason for this inconsistency could lie in the manufacturing process, which might have introduced irregularities in the composite’s structure. These irregularities can affect the bond quality between the fibers and DE, significantly impacting the overall performance of the actuators.

**Figure 7:**
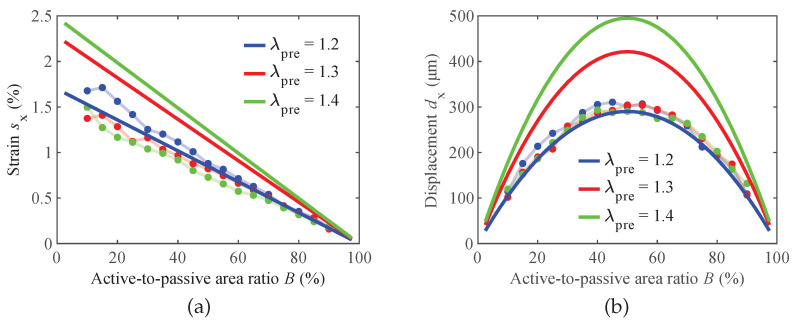
Comparison of experimental and simulation results for (**a**) strain $s_{x}$ and (**b**) displacement $d_{x}$, when compared to the lumped-parameter model at varying pre-stretches $\lambda_{pre}$, respectively.

Figure 7a presents the electrically induced electrode strain $s_{x}$ calculated according to Equation (6), where $d_{x}$ was computed in LTspice^®^ with the equivalent circuit model. Using the electro-mechanical analogies (Table 1), the magnitude values of the inductances $L_{active}$ and $L_{passive}$ were calculated from the experimentally determined Young’s moduli *Y* and initial thickness values $t_{DEA,initial}$ of the un-stretched composite actuators (as listed in Table 2). As observed in the experimental measurements, the strain exhibits a linear decrease as the active-to-passive ratio *B* increases. Furthermore, it is noteworthy that all the strain curves intersect at a common point, where a 100% active-to-passive ratio corresponds to a 0% strain. This intersection explains the convergence of the measured curves, providing insights into the actuation behavior as the active and passive areas vary.

Upon comparing the measured actuator that was subjected to a pre-stretch of 1.2 with the simulation results, good agreement is found between the two. However, the simulation model predicts an interesting phenomenon: an increase in actuation strain with higher pre-stretch levels. This observation is particularly evident in the simulated displacements illustrated in Figure 7b, where significantly higher absolute deformations are calculated for higher pre-stretch values. This finding suggests that the pre-stretch level plays a substantial role in influencing the actuation performance of the modeled DEA. However, this is not represented in the gathered experimental data, which are influenced comparably less by the pre-stretch.

Similar to the experimental measurements, the simulated displacement data also follow a parabolic curve, with the maximum deformation occurring at a 50% active-to-passive ratio, regardless of the pre-stretch level. This consistency in the displacement behavior shows the importance of maintaining a balanced 1:1 active-to-passive area ratio to achieve optimal actuation performance, irrespective of the pre-stretch applied. As with the simulation results for the strain, the calculated displacement overshoots the experimentally observed one.

## 5. Hyperelastic Model

### 5.1. Derivation of the Model

**Figure 8:**
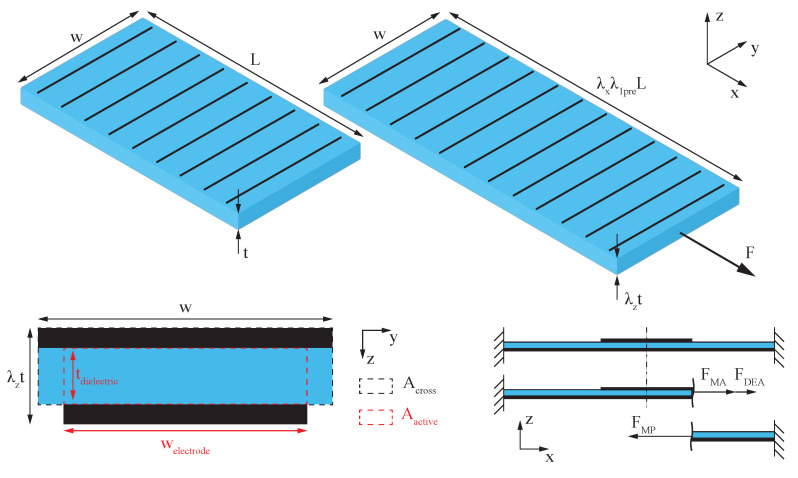
Schematic of the fiber-reinforced strip actuator, its dimensions, and the acting forces.

In contrast to the lumped-parameter model, the hyperelastic model captures the non-linear material properties of the composite strip and, therefore, should provide more accurate results, especially for large strains. Furthermore, the positive feedback loop of uniaxial strain and material thinning in the thickness direction on the electric field is taken into consideration. Figure 8 shows the forces acting in the *x*-direction during the measurement, which are represented in the equilibrium of forces,

(8) $$F_{MA}-F_{DEA}-F_{MP}=0.$$

Expressing the acting forces through the mechanical stress σ and the respective area leads to

(9) $$\sigma_{MA}-\sigma_{MP}-\frac{A_{active}}{A_{cross}}\sigma_{Maxwell}=0.$$

This approach includes the simplification of the uniform material properties throughout the whole cross-section of the actuator, which will lead to deviations in the model in material pairings with large differences in stiffness. The mechanical stress acting in the active and passive parts of the actuator strip is expressed through

(10) $$\begin{array}{cc} \sigma_{MA} & =\lambda_{A}\frac{\partial W(\lambda_{x}=\lambda_{A},\lambda_{y})}{\partial \lambda_{x}}, \end{array}$$

(11) $$\begin{array}{cc} \sigma_{MP} & =\lambda_{P}\frac{\partial W(\lambda_{x}=\lambda_{P},\lambda_{y})}{\partial \lambda_{x}}, \end{array}$$

where $W(\lambda_{x},\lambda_{y})$ is the Helmholtz free energy function of the hyperelastic material model, and $\lambda_{A}$ and $\lambda_{P}$ are the stretch ratios in the active and passive parts, respectively. To capture the mechanical material properties, the Yeoh model with

(12) $$\begin{array}{cc} W(\lambda_{x},\lambda_{y}) & =\sum_{i=1}^{3}C_{i}{\left(\lambda_{x}^{2}+\lambda_{y}^{2}+\lambda_{x}^{-2}\lambda_{y}^{-2}-3\right)}^{i} \end{array}$$

is used. The three material parameters $C_{1},C_{2}$, and $C_{3}$ are fit to the experimental data. The Maxwell stress acting in the active area of the cross-section can readily be calculated by

(13) $$\sigma_{Maxwell}=\varepsilon_{0}\varepsilon_{r}E^{2}=\varepsilon_{0}\varepsilon_{r}\frac{U_{HV}^{2}}{t^{2}\lambda_{z}^{2}}.$$

Inserting Equations (13), (10) and (11) into Equation (9) leads to

(14) $$\chi \varepsilon_{0}\varepsilon_{r}\frac{U_{HV}^{2}}{t^{2}}\lambda_{pre}\lambda_{a}^{2}=\lambda_{a}\frac{\partial W(\lambda_{x}=\lambda_{A},\lambda_{y})}{\partial \lambda_{x}}-\lambda_{p}\frac{\partial W(\lambda_{x}=\lambda_{P},\lambda_{y})}{\partial \lambda_{x}}$$

where $\chi =A_{active}/A_{cross}$ and $\lambda_{a}$ and $\lambda_{p}$ are the parts of the stretch ratios caused by the applied voltage $U_{HV}$. Superimposing the pre-stretch $\lambda_{pre}$ onto the electro-active stretches $\lambda_{a}$ and $\lambda_{p}$ leads to the true present stretch ratios $\lambda_{A}=\lambda_{pre}\lambda_{a}$ in the active part and $\lambda_{P}$ in the passive part, respectively. Due to the fixed length *l* of the actuator, the stretch ratios in the active and passive parts can be put into relation by

(15) $$\lambda_{a}l_{active}+\lambda_{p}l_{passive}=l.$$

This equation can be expressed with the active-to-passive ratio *B* through

(16) $$\lambda_{a}B+\lambda_{p}\left(1-B\right)=1.$$

Using this relationship, Equation (14) can be formulated as

(17) $$0=\lambda_{a}\frac{\partial W(\lambda_{x}=\lambda_{A},\lambda_{y})}{\partial \lambda_{x}}-\frac{1-\lambda_{a}B}{1-B}\frac{\partial W(\lambda_{x}=\lambda_{P},\lambda_{y})}{\partial \lambda_{x}}-\chi \varepsilon_{0}\varepsilon_{r}\frac{U^{2}}{t^{2}}\lambda_{pre}\lambda_{a}^{2}.$$

To solve the equation for $\lambda_{a}$ under different voltages and pre-stretch conditions, it is assumed that the fibers are blocking all deformation in the *y*-direction and hence that $\lambda_{y}=1$.

### 5.2. Comparison of the Hyperelastic Model Results

**Figure 9:**
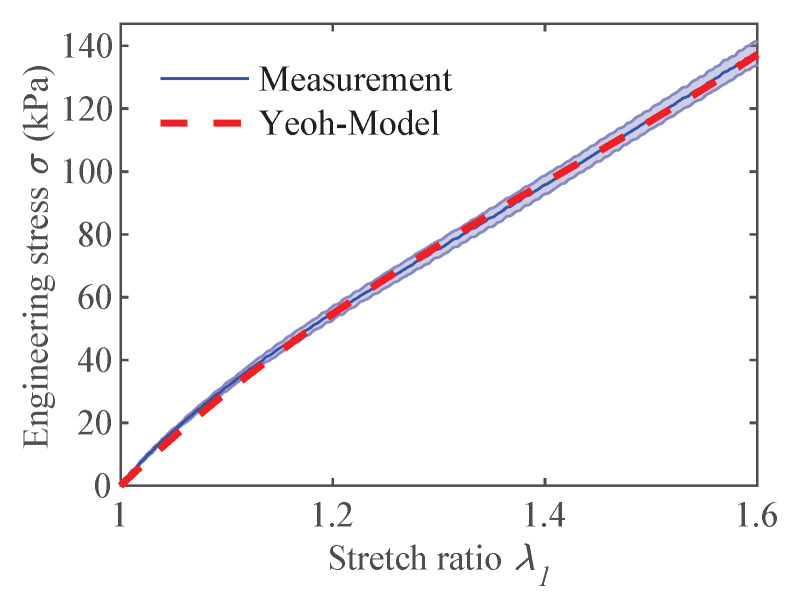
Stress–strain measurement of the fiber-reinforced DEA perpendicular to fiber direction and its fit to a hyperelastic Yeoh model.

To capture the hyperelastic properties of the material, the three-parameter Yeoh material model was used. The averaged tensile test results that were used to fit the material model in Ansys^®^ (Ansys^®^ Workbench, Release 19.0, Ansys, Inc., Canonsburg, PA, USA), as well as the resulting fit, are shown in Figure 9. The calculated model parameters are given in Table 3 and are used to compute the results of the hyperelastic model presented in Section 5.1.

**Figure 10:**
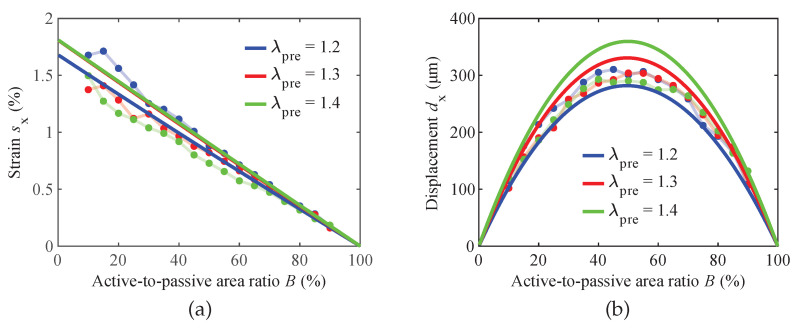
Comparison of experimental and simulation results for (**a**) strain $s_{x}$ and (**b**) displacement $d_{x}$, when compared to the hyperelastic model at varying pre-stretches $\lambda_{pre}$, respectively.

The predicted strains of the hyperelastic model, as shown in Figure 10a, exhibit the same trend of decreasing linearly from a 0% to 100% coverage ratio as the linear-elastic lumped-parameter model. However, contrary to the lumped-parameter model, the pre-stretch level is of smaller influence for the actuation strain. Taking into consideration the low sample numbers of the experiments, the predicted strain curves are within the expected deviations.

The parabolic shape seen both in the experiments and the lumped-parameter model is also present in the hyperelastic model, as illustrated in Figure 10b. As for the strain, the predicted displacements are in better agreement with the experimental results than in the lumped-parameter model. In contrast to the lumped-parameter model, the loss of tension in the passive area is also taken into consideration. In the case of $\lambda_{pre}=1.2$, this leads to a minor reduction in the theoretically optimal coverage ratio of 50% to around an optimum at around 49.5%. Although the influence is negligible in the study carried out here, if larger strains were to be investigated, the shift in the optimal coverage ratio would be more significant.

## 6. Discussion

The measurements of the electro-active strain (Figure 5b) reveal that incorporating uniaxially aligned stiff fibers into the DEA structure suppresses the actuation strain in the fiber direction and, hence, causes a unidirectional operating DEA. The experimental deformation evaluation of three fiber-reinforced strip actuators with different pre-stretches shows a linear decrease in electro-active strain with an increasing active-to-passive ratio. Contrary to the expectation that actuators with higher pre-stretch associated with a thickness reduction cause higher electro-active strains, actuators with higher pre-stretch show smaller electro-active strains in the experiment. The assumption that this is due to material stiffening could not be confirmed by subsequent tensile tests of the investigated DEAs. It is assumed that irregularities in the actuator manufacturing, which affect the bond quality between the fibers and DE, led to the observed behavior. The influence of the pre-stretch could not be consistently identified experimentally in this study and should be investigated in future work with a higher sample number and improved uniform fiber bonding. The displacement follows a downward opening parabolic curve, the maximum of which is in the active-to-passive ratio range between 40% and 55% for all pre-stretch levels.

The two analytical models show a comparable trend to the experimental data for electro-active strain and displacement. Moreover, both models confirm the hypothesis that the electro-active strain increases at higher pre-stretch ratios due to a thickness reduction in the DE. Independently of the pre-stretch, the models fulfill the boundary condition of zero strain at a coverage ratio of 100% and show a sharp maximum of displacement at an active-to-passive ratio of exactly 50%. Thus, our experimental and simulation results are in line with previous research carried out by Rosset et al. [14,15].

With respect to the experimental data, the lumped-parameter model shows very good agreement for a pre-stretch ratio of 1.2. However, for pre-stretch ratios of 1.3 and 1.4, the simulation with the hyperelastic model exhibits smaller deviations compared to the experimental data. Hence, neglecting the loss of tension phenomenon of the passive zone in the lumped-parameter model leads to a significant overshoot in the simulation values of the actuation strain and displacement (Figure 7).

Comparing the models, it is noteworthy that both underestimate the strain at a pre-stretch level of 1.2, whereas the opposite effect is observable for higher pre-stretches. A possible explanation could be that material effects, such as material softening over time, the dependence of the dielectric constant on strain, and the electrical properties of the soft electrodes, influence the results and are not considered in the models. As mentioned before, optimizing the fiber bonding and repeating the experiment with larger sample sizes could give further insights into this.

Both the experimental and simulation results show that the maximal displacement occurs for an active-to-passive ratio of 50%, even when varying the pre-stretch level. The parabolic displacement curves have a very small slope around this ratio compared to lower or higher coverage ratios. Thus, the sensitivity to DEA manufacturing irregularities and measurement uncertainties in electro-active strain measurement is lowest in this range. Therefore, it is recommended to investigate the actuation performance of planar operating DEAs that are clamped on both sides at an active-to-passive ratio of 50%. Considering the active-to-passive ratio range of 10% to 30% recommended by Carpi et al. [6] for the here studied rectangular DEAs, it should be noted that both the actuation strain and displacement are not constant in this ratio range. This underlines the fact that performance studies carried out with different active-to-passive ratios are not comparable even if the DE pre-stretch and DE film thickness are uniform. However, since we have shown experimentally and simulatively that the strain $s_{x}$ decreases linearly and is zero at a ratio of 100%, one could measure the strain at any desired coverage ratio and perform a linear curve fit. Then, the calculated actuation strain at an active-to-passive ratio of 0% could be used as a standardized comparison measure for DE membranes. In particular, this method can be applied to DEs in uniaxial DEAs with actuation strains of more than 100% (e.g., VHB), since, in this case, a 50% active-to-passive ratio does not provide the space for full-plane extension at actuation.

## 7. Conclusions

The key conclusion of our study is the importance of the active-to-passive ratio in dielectric elastomer actuators. Strains or displacements recorded at different active-to-passive ratios are not directly comparable. Therefore, we propose the calculation of a virtual electro-active strain for a coverage ratio of 0%, which is the maximum achievable strain. However, the displacement is greatest if the active and passive regions are of identical size, making a coverage ratio of 50% ideal for characterization and real-world applications.

## Figures and Tables

**Table 1:** Excerpt of electro-mechanical analogies for lumped systems.

Mechanical Quantity	Electrical Quantity
Force *F*	Current *i*
Velocity *v*	Voltage *u*
Compliance *n*	Inductance *L*

**Table 2:** Results of the mechanical characterization.

DEA	$\lambda_{\mathrm{pre}}$	*Y* (kPa)	$t_{\mathrm{DEA},\mathrm{inital}}$ (µm)
1	1.2	223	397
2	1.3	205	378
3	1.4	207	398

**Table 3:** Parameters of the hyperelastic Yeoh material model.

Yeoh Parameter	Fit Value (Pa)
$C_{1}$	54,407
$C_{2}$	−1778
$C_{3}$	2651

## Data Availability

The data presented in this study are available on request from the corresponding author.

## Abbreviations

The following abbreviations are used in this manuscript:

DEA	Dielctric elastomer actuator
DE	Dielectric elastomer
SPICE	Simulation program with integrated circuit emphasis
CNT	Carbon nanotube
PDMS	Polydimethylsiloxane
DIN	Deutsches Institut für Normung
